# Retention of Mutations in Colchicine-Induced Ornamental Succulent *Echeveria* ‘Peerless’

**DOI:** 10.3390/plants11243420

**Published:** 2022-12-07

**Authors:** Raisa Aone M. Cabahug, My Khanh Thi Ha Tran, Yun-Jae Ahn, Yoon-Jung Hwang

**Affiliations:** 1 Plant Genetics and Breeding Institute, Sahmyook University, Seoul 01795, Republic of Korea; 2 Department of Convergence Science, Sahmyook University, Seoul 01795, Republic of Korea; 3 Department of Horticultural Science, Kyungpook National University, Daegu 41566, Republic of Korea

**Keywords:** chemical mutation, ornamentals, plant mutagenesis, succulents

## Abstract

Mutation breeding has produced promising results, with exceptional attributes including pest/disease and environmental tolerance and desirable ornamental traits. Among the tools used in mutation breeding, chemical mutation is the most inexpensive way to develop novel plants. Succulents have gained popularity with high market demand because they require minimal watering and have plastic-like visuals. Ornamental succulents with rare leaf morphologies are costly. An LD_50_ study was conducted beforehand to determine the survival rates of colchicine-treated *Echeveria* ‘Peerless’. Mutants in the first generation (MV_1_) were identified and analyzed. Determining whether mutagenic characteristics are carried to the subsequent generation (MV_2_) is a key component in breeding programs. Additional investigation was performed by producing MV_2_ plants through vegetative propagation to determine mutagenic retention. For MV_2_, mutants exhibited shortened leaves, increased leaf width and thickness, and fewer leaves, which significantly differed from the control, indicating compactness, wider leaf apex, and varying leaf color. To confirm the mutations, stomatal analysis was conducted, wherein there was a decrease in density and an increase in stomatal size. Likewise, chromosome counting and flow cytometry analysis confirmed the induction of polyploidization. Colchicine induction to develop new cultivars with novel phenotypic and cytogenetic characters is suitable for ornamental succulents.

## 1. Introduction

A booming industry with an estimated annual growth rate of USD 500 million, floriculture is an important sector in agriculture, with a high demand to develop new varieties with improved attributes [1]. Growing in hype among millennials, succulent potted plants have been raved about and have increased market demand [2]. This is due to their beautiful geometric shapes, rosette-formed structures, unique leaf shapes, and most importantly, these plants require minimal care and watering, which fits the current lifestyle of busy metropolitan areas [3]. The peculiar leaf shape and color variation of succulents increase their ornamental value, thus demanding a higher selling price [4]. To produce these unique visual characteristics, natural breeding and cultural management practices are implemented; however, the former method requires years to develop, whereas the latter produces changes that may disappear under seasonal or environmental conditions (i.e., lighting conditions, watering, and temperature) [5].

To modify these plant characteristics and retain their unique features, the genetic information of a plant must be stably changed through mutagenesis [6]. Mutation breeding allows the induction of abrupt variations in the heritable DNA of a living cell or any organism without undergoing genetic recombination or natural breeding [7]. Among the tools used in mutation breeding, induced mutations using chemical mutagens are preferred because of their effectiveness (high mutation rates), ease of handling, and few detrimental effects. Additionally, this method produces long-lasting desirable changes compared with physical mutations that require sophisticated facilities [8,9].

Colchicine, an alkaline mitotic inhibitor, is a mutagen that has been reported to effectively induce morphological changes in ornamental plants such as chrysanthemum [10], gladiolus [11], marigolds [12], and orchids [13]. The mechanism by which colchicine induces compactness and change in color, size, and thickness of leaves and floral structure depends on its ability to inhibit microtubule formation and alter the chromosome number [14,15,16]. This process often results in polyploidization, which has also been found to exhibit such plant polymorphisms [17].

Our previous studies on the use of colchicine on leaf cuttings successfully produced putative succulent mutants using colchicine at certain concentrations and dipping durations [18]. This was conducted because there is scant information available regarding the attainable range and degree of effects of chemical mutagenesis on asexually propagated succulent species or cultivars. The results of our previous study suggested that colchicine treatments induced compactness in plants, which exhibited fewer, shorter but thickened leaves compared with those of the control. In addition, stomatal changes were also evident, as less dense stomates were found with increased stomatal size. Genome size evaluation using flow cytometry also revealed mixoploidy in these putative plants. These observed changes were also reported by other researchers using colchicine and have been attributed to polyploidization for both phenotypic and plant organ traits, as well as stomatal characteristics [19,20].

The question remains as to whether the retention of induced mutations brought about by mutagenesis in the first generation (MV_1_) will be carried on to its subsequent generation (MV_2_). Hence, this study was conducted to evaluate the mutagenic effects of colchicine on selected *Echeveria* ‘Peerless’ from MV_1_ to MV_2_ at both the phenotypic and cytogenetic levels.

## 2. Results and Discussion

During screening for mutants, Eng and Ho [21] suggested that the use of indirect (i.e., morphological and physiological traits, including plant measurements and stomatal evaluation) and direct (i.e., chromosome counting and flow cytometry) methods is needed. However, the latter method is preferred because of its accuracy, uninfluenced by environmental conditions, and high reliability. Our study used both direct and indirect methods to evaluate the retention of mutagenic changes in the primary generation of colchicine-treated *Echeveria* ’Peerless’.

### 2.1. Phenotypic Traits

To determine phenotypic differences, plant and leaf parameters were evaluated to assess the growth and development of mutant plants. Likewise, lamellae color was measured and analyzed through spectrophotometric color readings using the CIELAB color space.

#### 2.1.1. Plant Growth Parameters

Parameters of plant and leaf measurements are shown in Table 1 for *Echeveria* ‘Peerless’ for two successive generations. Data on mutant plant growth and development showed varied effects across treatments; however, there was a clear difference between the control plants and the putative mutants.

**Figure 1:**
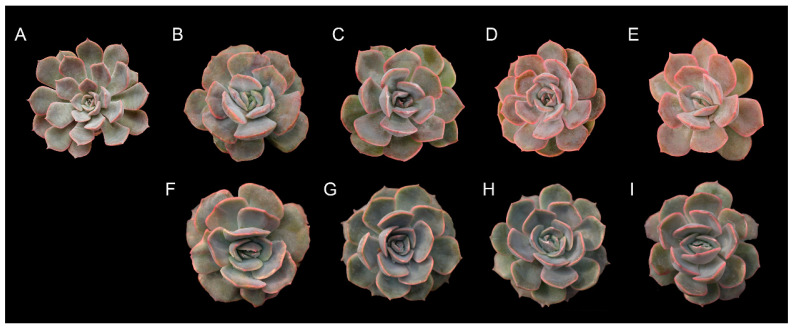
*Echeveria* ‘Peerless’ plants treated with colchicine: (**A**) control at MV_1_ (**B**) 0.2% + 3 h, (**C**) 0.4% + 3 h, (**D**) 0.6% + 3 h, and (**E**) 0.8% + 3 h and at MV_2_ (**F**) 0.2% + 3 h, (**G**) 0.4% + 3 h, (**H**) 0.6% + 3 h, and (**I**) 0.8% + 3 h.

In both generations, the results indicate that colchicine-treated succulents were generally taller plants with shorter, wider, and thicker leaves and fewer than those of the control (Figure 1).

**Figure 2:**
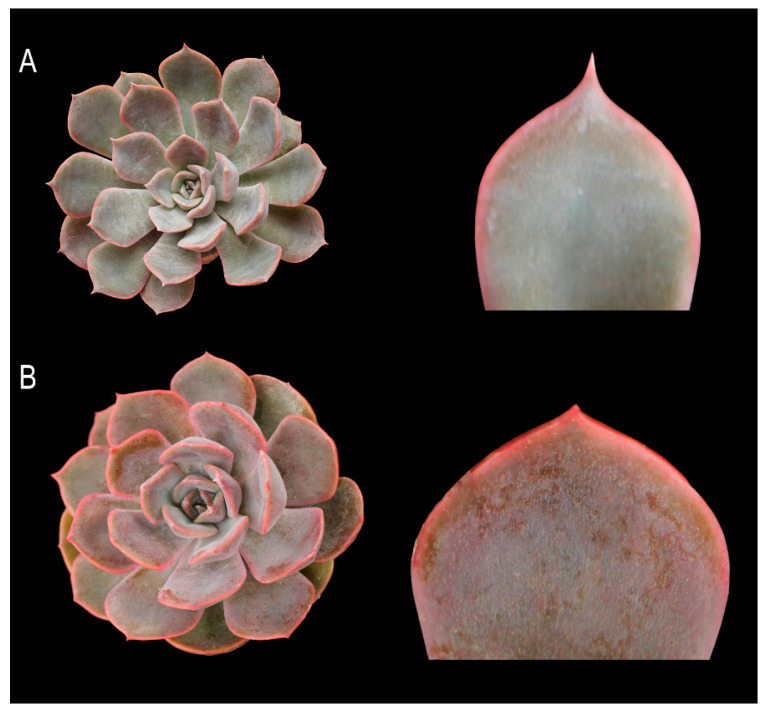
Effects of colchicine on *E.* ‘Peerless’ showing the (**A**) control and (**B**) mutant whole plant and leaf apex.

These developmental differences in leaf measurements reflected equivalent changes in leaf shape and apex. Figure 2 highlights the differences between control and mutant plants, wherein the leaves were altered from being cuneate in the control to being obovate in the mutants with much wider and less prominent tips.

Several studies have reported that colchicine-induced mutants among ornamental crops, such as calendula, chrysanthemums, gladiolus, and petunia [15,19,22,23], exhibited thicker leaves, darker colors in their leaves and flowers, and high compactness, which were attributed to polyploidy plants [17].

Polyploids have been found to possess more advantageous features than their diploid counterparts. They demonstrate superior environmental adaptability, disease resistance, and prominent features [24]. These changes are associated with the application of colchicine as an effective mutagen that prevents the formation of microtubules and spindle fibers, which in turn leads to chromosome doubling and polyploid induction [25,26]. The compactness exhibited in *E.* ‘Peerless’ may be due to ‘high-ploidy syndrome’, in which the mutants display enhanced cell expansion but reduced cell division or slower growth rates [27].

#### 2.1.2. Leaf Spectrophotometric Color Readings

The color of the lamellae of the plants was also evaluated. Spectrophotometric color readings were obtained using a spectrophotometer that uses the L*a*b* color spaces to indicate lightness, hue, and saturation, respectively. One-way analysis of variance (ANOVA) was used to determine significant differences in the mean CIELAB scores (L*, a*, and b*) (Table 2).

Colchicine treatment did not significantly affect the L* and b* values. However, it significantly affected a* values (*p <* 0.01) in *E.* ‘Peerless’ MV_1_ plants. This shows that the control plants had a more dominant red hue than the mutant plants.

In the subsequent generations, all color spaces were found to be significantly affected by colchicine treatment (*p <* 0.05). Lighter colors were observed in treated plants with a lesser red hue and less saturation than in the control.

These types of color changes in the leaves of colchicine-treated mutants have often been attributed to abnormalities in chlorine production in the gladiolus [19], as this micronutrient is actively involved in photosynthesis [28], turgor regulation [29], and elongation and growth [30]. Conversely, other studies have suggested that reduced chlorophyll content in colchicine-treated plants may be due to structural modification of the lamellar or thylakoid membranes of the chloroplast [31].

The retention of phenotypic traits from MV_1_ to MV_2_ may be attributed to the stability of the mutation in the subsequent generation. Similarly, upon induced polyploidy in the first generation, the use of vegetative propagation allows fixation and maintenance of the desired genotype under domestication, which has been commonly used in numerous ornamentals [32].

### 2.2. Stomata Characteristics

Results show that colchicine treatment significantly affected the stomatal characteristics of *E.* ‘Peerless’ for both generations (*p ≤* 0.001), as shown in Table 3.

**Figure 3:**
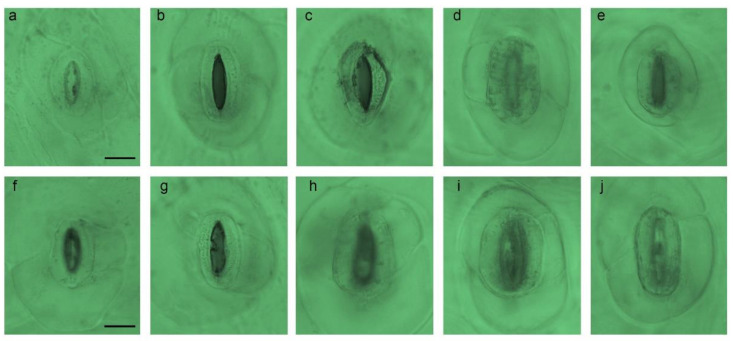
Stomata characteristics of colchicine-induced *E.* ‘Peerless’, showing the (**a**,**f**) control at MV_1_ (**b**) 0.2% + 3 h; (**c**) 0.4% + 3 h; (**d**) 0.6% + 3 h; and (**e**) 0.8% + 3 h and at MV_2_ (**g**) 0.2% + 3 h; (**h**) 0.4% + 3 h; (**i**) 0.6% + 3 h; and (**j**) 0.8% + 3 h. Scale bar = 10 µm.

For both successive generations, the stomatal density decreased in the colchicine-induced mutants with increased stomatal size, wherein control plants showed the smallest stomatal size. Additionally, higher concentrations (0.6% and 0.8%) resulted in larger stomata (Figure 3).

Manzoor et al. [17,19] emphasized that these changes in stomatal size are commonly observed in mutagen-induced polyploids. Likewise, determining stomatal changes, especially identifying an increase in size, has often been used as an indicator of putative mutants, which is a simple and non-destructive method for mutant screening [33,34].

As polyploids incite cell enlargement, stomatal dimensions are equally affected. Cells carrying larger genomic material ultimately grow to retain a constant ratio between the nuclear and cytoplasmic volume, which then enhances the expression of proteins brought about by the increase in the gene number [35]. Similar results of enlarged stomata and decreased density have been found in colchicine-induced horticultural and agronomic crops, such as *Stevia rebaudiana* Bertoni [36], *Gladiolus grandifloras* [19], *Glycyrrhiza glabra* [33], and African marigolds [37]. Consistent trends for stomatal data for successive generations suggested that mutagenic characteristics were retained from the MV_1_ stage.

### 2.3. Chromosome Counting and Nuclear DNA Genome Size Estimation Using Flow Cytometry

The Crassulaceae family possesses very small chromosomes, within which the *Echeveria* genus has been reported to have a great diversity of larger gametic numbers ranging from *n* = 12–34 to *n* = 260 [38,39]. Because of the wide range of chromosome numbers in both n and 2 n, a few species that were identified as diploids could possess chromosome numbers as high as those of polyploids [39,40]. However, there have been no reports of chromosome counting in cultivars circulating in succulent or ornamental markets.

In this study, *E.* ‘Peerless’ control and putative plants were subjected to chromosome counting (Table 4). However, among the mutant groups, only those with lower concentrations (0.2% and 0.4%) produced young roots for mitotic chromosome counting. This may be due to the slow growth reported for chemically induced polyploids.

**Figure 4:**
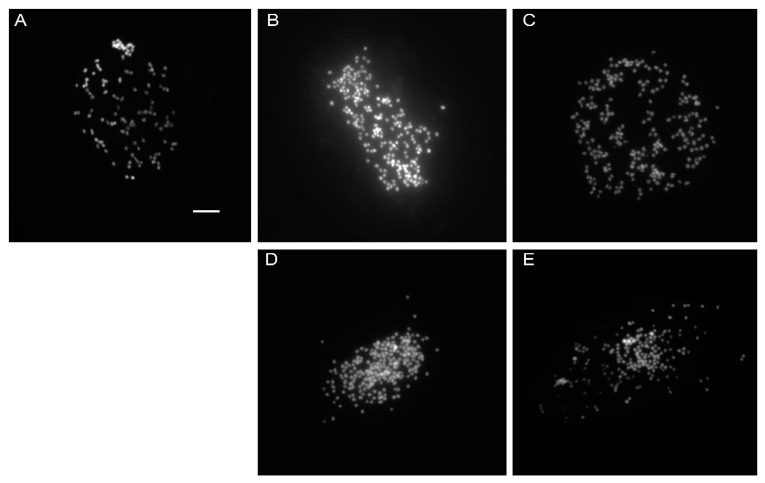
*Echeveria* ‘Peerless’ chromosomes with DAPI staining: (**A**) control, MV_1_ (**B**) 0.2% + 3 h and (**C**) 0.4% + 3 h and MV_2_ (**D**) 0.2% + 3 h and (**E**) 0.4% + 3 h.

Chromosome counts from the control plants yielded 2 n = > 110, while those of the colchicine-treated plants in both generations were almost twice the number at MV_1_ (2 n = > 204–206) and MV_2_ (2 n = > 184–202) (Figure 4). In MV_1_, the chromosome number was found to be a proportionally higher number at higher concentration, given that at 0.4% it had 2 n= > 215, followed by those at 0.2% with 2 n = > 206. Some studies have reported that some *Echeveria* species possess as high as *6 x*–*10 x* ploidy levels, with about a 2 n = 260~270 chromosome number [39,40,41].

It is probable that there was a slight decrease in the number of chromosomes in MV_2._ However, based on the retained morphological expression, this had little to no effect on the compactness of the plants. However, other qualitative characteristics such as disease resistance or environmental adaptability may be affected and should be investigated at a later stage. Likewise, several reports have shown that chromosomal instability is recognized in polyploids [42,43]. On the other hand, the study of Pierre et al. [44] reported chromosome losses among chemically induced polyploids, but they became stable over time. 

The karyotype indicated in the chromosome count successfully identified chromosome doubling. Chromosome doubling or, as it is often referred to, ‘polyploidization’, is often pointed out as the root cause for the increase in biomass yield [27] and phenotypic changes [17], which play a vital role in cultivar development as well as normal plant growth and development. Zhou et al. [45] reported that the application of colchicine in plants restrains microtubules and inhibits gene expression of cytokinesis, which leads to slow cell activity, which would explain the slow plant growth observed in *E.* ‘Peerless’.

Flow cytometry (FCM) is a reliable tool for analyzing the genome sizes of various plant species as well as a screening method for putative mutants and cultivars using regenerated young plant tissues [46]. The use of flow cytometry to estimate the DNA genome size of *E.* ‘Peerless’ significantly shows and supports the chromosome doubling found in the chromosome counting (Table 5). Compared with the control (1.06 pg), plants within the mutant groups for both the subsequent generations had significantly doubled genome sizes.

**Figure 5:**
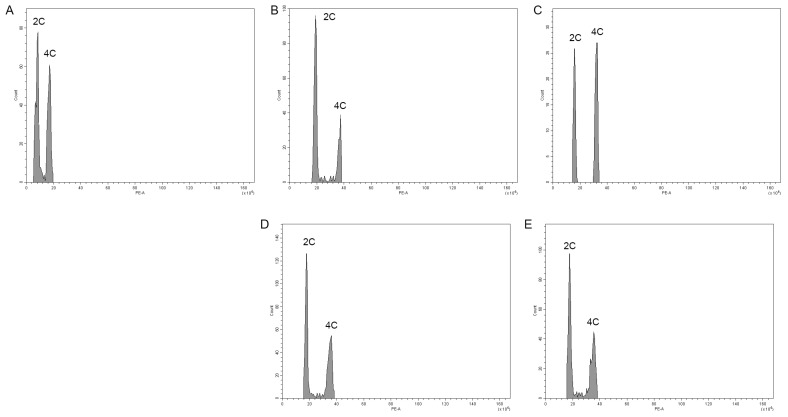
Estimation of the nuclear DNA content of *Echeveria* ‘Peerless’: (**A**) control, MV_1_ (**B**) 0.2% + 3 h and (**C**) 0.4% + 3 h and MV_2_ (**D**) 0.2% + 3 h and (**E**) 0.4% + 3 h, showing corresponding peaks to nuclei 2 C and 4 C with an external standard test using *Glycine max* cv ‘Polanka’. The *Y*—axis of each figure indicates the *count* of nuclei, while the *X*—axis indicates the PE-A or propidium iodide fluorescence intensity.

As seen in the histogram of the FCM (Figure 5), 2 C peaks of the control (A) were found in channel 8, while the mutant group 2 C peaks were found further away in channels 16 to 20, indicating a significant peak difference in the genome size estimates. This includes both results for the succeeding generations. It was also observed that the dip in the chromosome number for MV_1_ to MV_2_ was identified using FCM. However, the differences in the chromosome number within the mutant group were not as significant as those in the control group. Similar results of increased nuclear DNA content are found in colchicine-induced plants, such as *Platanus acerifolia* [47], *Gerbera* lines [48], and *Catharanthus roseus* [49], which confirms that the analyzed plants exhibit polyploidy.

## 3. Materials and Methods

### 3.1. Plant Materials

Putative mutants from 3 h of treatment at various colchicine concentrations (0.2%, 0.4%, 0.6%, and 0.8%) from a previous study were evaluated for the retention of mutagenic traits. The *E.* ‘Peerless’ mutants were grown in a succulent nursery in Goyang City, Gyeonggi-do Province, South Korea.

**Figure 6:**
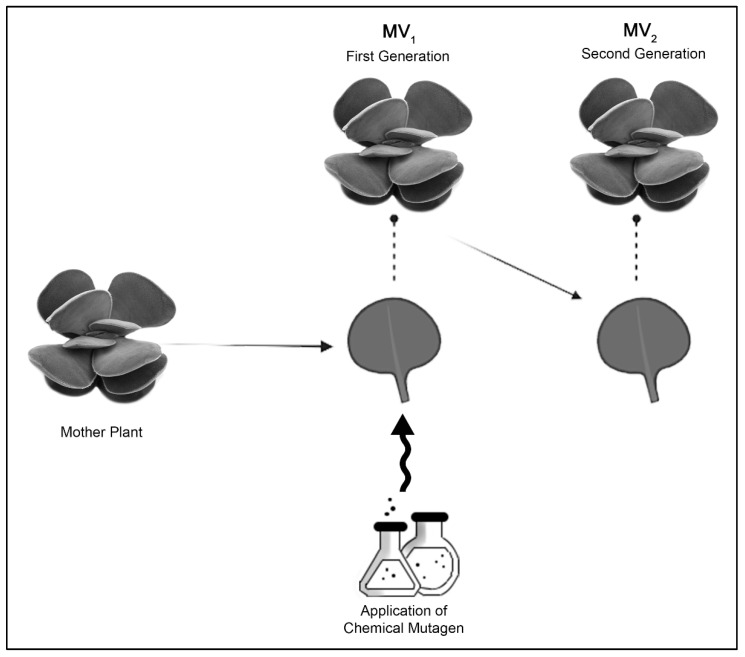
Diagram showing the procedure for chemical treatment and generation of MV_1_–MV_2_ through vegetative propagation.

Surviving mutant plants were considered MV_1_. Vegetative propagation of leaf cuttings from the three lower whorls (location of the mature leaves) was collected from succulent mutant plants and planted, and the resulting progenies were considered MV_2_ (Figure 6).

### 3.2. Plant Management

All the plants were watered once per week. Dead leaf cuttings were removed to avoid possible infection to other growing leaf cuttings from the decaying ones. The average temperature was 24 ± 3 °C, and the relative humidity was 65–70%.

### 3.3. Data Collection

#### 3.3.1. Phenotypic Evaluation

Plant (height, diameter, and number of leaves) and leaf (length, width, and thickness) growth parameters were measured, and morphological characteristics (leaf shape and apex) were observed. The CIELAB color readings were obtained using a handheld spectrophotometer (CM−2600 d; Konica Minolta Inc., Tokyo, Japan).

#### 3.3.2. Stomatal Characteristics

Putative plants were sampled for stomatal evaluation by collecting leaves near the base whorl. The nail varnish technique was used to evaluate the stomatal size and density [50]. Microscopic observations of stomatal impression slides were performed under a light microscope (Olympus BX53 F, Tokyo, Japan) at 40× and 80× magnifications. To determine the stomatal density, three counts were obtained per leaf at three adjacent locations across the surface. The stomatal size was measured using ImageJ software (v. 1.52 a, National Institutes of Health, Bethesda, MD, USA).

#### 3.3.3. Chromosome Counting

Young roots were harvested from in vivo grown mutant *E.* ‘Peerless’ under ice-cold water and were treated with 2 mM 8-hydroxyquinoline for 5 h at 25 °C. Carnoy’s solution (3:1 acetic acid–ethanol) was used to fix the roots overnight at room temperature and stored in 70% ethanol at 4 °C to preserve the roots. The fixed root tips were washed with distilled water prior to enzyme treatment (0.3% cellulose, cytohelicase, and pectolyase) at 37 °C for 90 min. The enzyme-treated roots were transferred to a 1.5 mL tube containing Carnoy’s solution and vortexed for 20 s. The homogenized root meristems were placed on ice for 5 min and centrifuged at 13,000 rpm to collect pellets. The supernatant was discarded, and the pellet was immediately resuspended in acetic acid–ethanol (9:1) solution. The final suspension was spread on a 70 °C pre-warmed glass slide using the steam drop method [51].

#### 3.3.4. Flow Cytometry Analysis

Flow cytometry analysis was performed based on the methods of Dolezel et al. [52]. Healthy young leaves (approximately 20 mg) from the putative mutants were chopped using a sharp razor blade and put on a petri dish, and 1 mL of ice-cold nuclei isolation buffer was added. The homogenate was mixed by pipetting up and down the solution to avoid air bubbles. The mixture was then filtered through a 50 µm nylon mesh and further filtered through a 30 µm nylon mesh (CellTrics Filters, Sysmex Asia Pacific, Singapore). The final homogenate was labeled and 50 µL propidium iodide (Sigma-Aldrich, St. Louis, MO, USA, cat. no. P4170; Molecular Probes; cat. no. P3566) was simultaneously added to 50 µL RNase (Sigma-Aldrich, St. Louis, MO, USA, cat. no. R5000) and placed in a 1.5 mL Eppendorf tube (Sigma-Aldrich, St. Louis, MO, USA, T9786-1000EA).

Thereafter, the tube was placed in a CytoFLEX flow cytometer equipped with a 50 mW 488 nm solid-state diode laser (Beckman Coulter Inc., Pasadena, CA, USA). An external method of DNA content analysis was employed, which involved successive analysis of the sample and the standard (*Glycine max* cv ‘Polanka’) [53]. The peaks of both the standard and sample were compared using CytExpert v2.3 software (Beckman Coulter Inc., Pasadena, CA, USA), and the sample DNA content was computed.

#### 3.3.5. Statistical Analysis

Numerical data obtained from the morpho-anatomical evaluation were subjected to analysis of variance (ANOVA). Significant differences between means were analyzed using Duncan’s multiple range test (DMRT) at a 5% significance level. All statistical analyses were performed using SPSS version 22 (IBM Corp., Armonk, NY, USA).

## 4. Conclusions

Satisfying all methods for confirming polyploidization for both successive generations of colchicine-induced *E.* ‘Peerless’ was undertaken in this study. Along with the phenotypic and stomatal traits, combined with a more intensive method in chromosome counting and FCM analysis, colchicine-induced *E.* ‘Peerless’ mutants were found to successfully retain their mutagenic characteristics in the succeeding generation through vegetative propagation. Through chemical mutagenesis, these mutants were found to possess novel genetic and phenotypic variations. This convenient and efficient method of creating new cultivars with high ornamental value will demand higher market prices and increased revenue for succulent breeders, nurseries, and other key players in the ornamental industry, as well as other related plant species.

## Figures and Tables

**Table 1:** Plant and leaf growth parameters of MV_1_ and MV_2_ *E.* ‘Peerless’ mutants treated with colchicine treatment.

Treatment	MV_1_						MV_2_					
	Plant (mm)			Leaf (mm)			Plant (mm)			Leaf (mm)		
	Height	Diameter	No. of Leaves	Length	Width	Thickness	Height	Diameter	No. of Leaves	Length	Width	Thickness
Control	35.53 ± 4.92 ^1^	97.75 ± 5.15	33.67 ± 0.88	39.04 ± 7.44	21.23 ± 3.95	5.21 ± 0.91	36.32 ± 1.24	65.15 ± 1.20	49.00 ± 0.84	45.05 ± 0.83	19.18 ± 0.10	4.98 ± 0.12
0.2% + 3 h	33.58 ± 6.46	73.94 ± 3.53	18.50 ± 1.50	34.67 ± 1.05	31.38 ± 1.14	5.84 ± 4.21	48.45 ± 0.00	85.88 ± 0.00	30.00 ± 0.00	41.96 ± 0.00	29.97 ± 0.00	9.82 ± 0.00
0.4% + 3 h	42.60 ± 9.25	85.43 ± 5.15	20.00 ± 2.08	37.98 ± 3.58	23.70 ± 4.96	7.55 ± 1.26	49.57 ± 1.20	83.81 ± 0.83	29.00 ± 1.41	33.96 ± 2.23	26.97 ± 1.36	9.59 ± 1.14
0.6% + 3 h	48.92 ± 4.11	86.79 ± 2.68	19.67 ± 2.00	38.17 ± 3.59	23.98 ± 4.35	8.35 ± 2.13	55.95 ± 0.00	90.42 ± 0.00	39.00 ± 0.00	41.56 ± 0.00	26.27 ± 0.00	9.53 ± 0.00
0.8% + 3 h	36.64 ± 6.00	65.21 ± 2.46	22.40 ± 1.87	30.43 ± 3.85	22.12 ± 1.39	23.1 ± 1.17	50.62 ± 0.71	73.34 ± 2.07	25.80 ± 1.38	36.26 ± 0.96	24.83 ± 0.74	8.80 ± 0.61

^1^ Data are presented as Mean ± Standard Error (SE).

**Table 2:** CIELAB color values of colchicine-induced MV_1_–MV_2_ mutant *E.* ‘Peerless’.

Treatment	MV_1_			MV_2_		
	L*	a*	b*	L*	a*	b*
Control	40.73 ± 1.60 ^1^	6.48 ± 1.08 a ^2^	12.19 ± 2.35	39.13 ± 1.53 d	5.52 ± 2.86 a	13.19 ± 2.80 a
0.2% + 3 h	47.60 ± 1.48	2.15 ± 0.48 c	6.43 ± 0.64	48.84 ± 2.66 c	5.07 ± 0.92 a	6.72 ± 0.58 b
0.4% + 3 h	42.92 ± 3.36	2.00 ± 0.72 c	11.91 ± 1.59	49.23 ± 1.12 c	3.28 ± 0.68 b	7.90 ± 1.07 b
0.6% + 3 h	41.18 ± 2.02	−0.81 ± 0.44 e	11.79 ± 2.93	50.32 ± 0.54 b	5.09 ± 0.62 a	6.45 ± 1.12 b
0.8% + 3 h	45.58 ± 2.85	0.51 ± 1.29 d	10.50 ± 1.27	53.60 ± 1.54 b	2.87 ± 0.24 c	7.01 ± 0.77 b
F-test ^2^	NS	**	NS	*	*	*

^1^ Data are presented as Mean ± Std Dev. ^2^ Columns with the same letters are not significantly different by Duncan’s multiple range test at *p* = 0.05. NS, *, **, non-significant or significant at *p* = 0.05, or 0.01, respectively.

**Table 3:** Stomatal data on the adaxial area of control and MV_1_–MV_2_ *E.* ‘Peerless’ mutants treated with colchicine.

Treatment	MV_1_		MV_2_	
	Density	Size (µm)	Density	Size (µm)
Control	10.33 ± 0.33 ^1^ a ^2^	20.58 ± 0.59 d	10.54 ± 0.81 a	19.26 ± 0.35 d
0.2% + 3 h	9.33 ± 0.88 b	25.68 ± 0.74 b	7.67 ± 0.67 b	24.02 ± 0.14 c
0.4% + 3 h	6.33 ± 0.33 c	25.92 ± 0.67 b	5.67 ± 0.67 c	27.31 ± 0.14 b
0.6% + 3 h	8.35 ± 0.89 b	28.03 ± 0.25 a	9.00 ± 0.00 b	28.10 ± 0.03 a
0.8% + 3 h	8.55 ± 0.67 b	22.75 ± 0.15 c	8.33 ± 0.33 b	28.47 ± 0.12 a
F-test ^3^	**	**	**	**

^1^ Data are presented as Mean ± Std Dev. ^2^ Columns with the same letters are not significantly different by Duncan’s multiple range test at *p* = 0.05. ^3^ NS, **, non-significant or significant at *p* = 0.05, or 0.01, respectively.

**Table 4:** Chromosome number of colchicine-induced *E.* ‘Peerless’.

Treatments	MV_1_	MV_2_
Control	2 n = > 110	
0.2% + 3 h	2 n = > 206	2 n = > 204
0.4% + 3 h	2 n = > 215	2 n = > 184

**Table 5:** Nuclear DNA Genome size estimate of *E.* ‘Peerless’.

Treatments	2 C (Mbps) ^1^	CV (%) ^2^	1 C (Mbps)	1 C (pg) ^3^
Control	2071.63	0.72	1035.82	1.06
MV_1_				
0.2% + 3 h	4949.46	1.04	2474.73	2.53
0.4% + 3 h	4110.86	1.67	2055.43	2.10
MV_2_				
0.2% + 3 h	4604.74	0.48	2302.37	2.35
0.4% + 3 h	4618.33	1.30	2309.17	2.36

^1^ Mbps—megabase pairs. ^2^ CV = coefficient of variation (%); reliable results should be <5%. ^3^ pg—picograms.

## Data Availability

The authors confirm that the data supporting the findings of this study are available within the article.
